# Store-Operated Ca^2+^ Entry Does Not Control Proliferation in Primary Cultures of Human Metastatic Renal Cellular Carcinoma

**DOI:** 10.1155/2014/739494

**Published:** 2014-07-09

**Authors:** Silvia Dragoni, Ilaria Turin, Umberto Laforenza, Duilio Michele Potenza, Cinzia Bottino, Toma N. Glasnov, Martina Prestia, Federica Ferulli, Anna Saitta, Alessandra Mosca, Germano Guerra, Vittorio Rosti, Ombretta Luinetti, Carlo Ganini, Camillo Porta, Paolo Pedrazzoli, Franco Tanzi, Daniela Montagna, Francesco Moccia

**Affiliations:** ^1^Department of Biology and Biotechnology “Lazzaro Spallanzani,” University of Pavia, Via Forlanini 6, 27100 Pavia, Italy; ^2^Laboratory of Immunology Transplantation, Foundation IRCCS Policlinico San Matteo, Piazzale Golgi 19, 27100 Pavia, Italy; ^3^Department of Molecular Medicine, University of Pavia, Via Forlanini 6, 27100 Pavia, Italy; ^4^Christian Doppler Laboratory for Flow Chemistry, Institute of Chemistry, Karl-Franzens-University Graz, Heinrichstrasse 28, 8010 Graz, Austria; ^5^Medical Oncology, “Maggiore della Carità” University Hospital, 28100 Novara, Italy; ^6^Department of Health Sciences, University of Molise, Via F. de Santis, 86100 Campobasso, Italy; ^7^Laboratory of Biotechnology, Center for the Study of Myelofibrosis, Foundation IRCCS Policlinico San Matteo, Piazzale Golgi 19, 27100 Pavia, Italy; ^8^Department of Pathological Anatomy, Foundation IRCCS Policlinico San Matteo, Piazzale Golgi 19, 27100 Pavia, Italy; ^9^Medical Oncology, Foundation IRCCS Policlinico San Matteo, Piazzale Golgi 19, 27100 Pavia, Italy; ^10^Department of Clinical-Surgical, Diagnostic and Pediatric Sciences, University of Pavia, 27100 Pavia, Italy; ^11^Department of Physiology, University of Pavia, Via Forlanini 6, 27100 Pavia, Italy

## Abstract

Store-operated Ca^2+^ entry (SOCE) is activated following depletion of the inositol-1,4,5-trisphosphate (InsP_3_)-sensitive Ca^2+^ pool to regulate proliferation in immortalized cell lines established from either primary or metastatic lesions. The molecular nature of SOCE may involve both Stim1, which senses Ca^2+^ levels within the endoplasmic reticulum (ER) Ca^2+^ reservoir, and a number of a Ca^2+^-permeable channels on the plasma membrane, including Orai1, Orai3, and members of the canonical transient receptor (TRPC1–7) family of ion channels. The present study was undertaken to assess whether SOCE is expressed and controls proliferation in primary cultures isolated from secondary lesions of heavily pretreated metastatic renal cell carcinoma (mRCC) patients. SOCE was induced following pharmacological depletion of the ER Ca^2+^ store, but not by InsP_3_-dependent Ca^2+^ release. Metastatic RCC cells express Stim1-2, Orai1–3, and TRPC1–7 transcripts and proteins. In these cells, SOCE was insensitive to BTP-2, 10 *µ*M Gd^3+^ and Pyr6, while it was inhibited by 100 *µ*M Gd^3+^, 2-APB, and carboxyamidotriazole (CAI). Neither Gd^3+^ nor 2-APB or CAI impaired mRCC cell proliferation. Consistently, no detectable Ca^2+^ signal was elicited by growth factor stimulation. Therefore, a functional SOCE is expressed but does not control proliferation of mRCC cells isolated from patients resistant to multikinase inhibitors.

## 1. Introduction

Renal cell carcinoma (RCC) is by far the most common kidney cancer, accounting for 2%-3% of all adult malignancies and representing the 7th most common cancer in men and the 9th most common cancer in women [1]. The only treatment for patients with localized RCC is radical surgical resection of the tumour; however, 20–30% of patients present with synchronous metastases at diagnosis, and up to 30–35% of more patients, initially radically resected, will eventually develop metastases over time [2]. Targeting the intricate vascular network which develops around the primary tumour and is the gateway for metastasis dissemination has emerged as the most active treatment strategy to fight metastatic RCC (mRCC) [3–7]. In the past 7-8 years, the development and registration of molecularly targeted agents, inhibiting either the vascular endothelial growth factor (VEGF)/VEGF(Rs) pathway or mammalian target of rapamycin (mTOR), have dramatically improved the prognosis of mRCC patients [6, 7]. However, despite this abundance of active reagents, progression-free survival (PSF) for the majority of these patients rarely exceeds eleven months [6]. This is due to the ultimate development of drug resistance in the majority of patients initially responsive to treatment (3-4) and to the presence of so-called primary refractory patients (i.e., those who do not respond from the very beginning) [8]. There is, therefore, an unmet demand for discovering alternative molecular targets to achieve the complete regression of spreading metastases in both resistant and refractory mRCC patients, as well as in those experiencing treatment-related adverse events.

Store-operated Ca^2+^ entry (SOCE) is a ubiquitous mechanism which plays a key role in several key steps to malignant transformation, such as cell proliferation, migration, and metastatization [9–14]. A variety of molecular mechanisms have been proposed to sustain SOCE [4, 15]. Both G-protein coupled receptors (GPCRs) and tyrosine kinase receptors (TKRs) engage distinct isoforms of phospholipase C (PLC), that is, PLC*β* and PLC*γ* respectively, to cleave phosphatidylinositol 4,5-bisphosphate, into inositol-1,4,5-trisphosphate (InsP_3_) and diacylglycerol (DAG). InsP_3_ induces a massive Ca^2+^ release from the endoplasmic reticulum (ER), the most abundant intracellular Ca^2+^ reservoir, by activating the so-called InsP_3_ receptors (InsP_3_Rs). The consequent fall in intraluminal Ca^2+^ levels is detected by the ER Ca^2+^ sensor, Stim1 [4, 16]. Once activated, Stim1 proteins aggregate and migrate towards subplasmalemmal* puncta*, where they tether to and gate the Ca^2+^-selective channel Orai1 [4, 13]. Stim1 and Orai1-mediated SOCE control cell cycle progression and metastatization in several human malignancies, including those of glia [17], liver [18, 19], breast, and uterine cervix [20]. Conversely, no functional role has so far been established for Stim1 and Orai1 paralogues, that is, Stim2 and Orai2, in carcinogenesis, while Orai3 is recruited by Stim1 to activate SOCE and cell proliferation in some oestrogen receptor-negative human breast cancer lines [21]. Alternatively, Stim1 might trigger SOCE by conveying the ER Ca^2+^ load to nonselective Ca^2+^-permeable pathways, such as those belonging to the canonical transient receptor (TRPC1-7) family of ion channels [15]. Stim1 may bind to and activate TRPC1, TRPC4, and TRPC5; in addition, Stim1 causes TRPC3 and TRPC6 to mediate SOCE by mediating the heteromultimerization of TRPC3 with TRPC1 and of TRPC6 with TRPC5 [15]. TRPC1 and TRPC6 have convincingly linked to SOCE in a growing number of human tumours, including breast carcinoma [22], glioblastoma multiforme (GBM) [23, 24], and hepatoma [18, 19]. These results put forward SOCE as an alternative target to devise novel antimetastatic treatments [10, 12, 14, 25]. With some notable exceptions [17], however, the physiological function of SOCE has been characterized in immortalized cell lines established from either primary or metastatic lesions that do not accurately recapitulate the heterogeneity and complexity of human neoplasms. This issue is particularly relevant when seeking alternative strategies to fully eradicate metastases by inhibiting SOCE. Accordingly, earlier work showed that oncogenesis reduces the need for Ca^2+^ entry to initiate DNA synthesis and cell replication, a phenomenon known as “habituation” to extracellular Ca^2+^ [26, 27].

We have recently demonstrated that Stim1, Orai1, and TRPC1 mediate SOCE and control proliferation in circulating endothelial progenitor cells (EPCs) isolated from untreated mRCC patients [10]. The present study aimed to assess whether a functional store-dependent Ca^2+^ inflow is present and drives cell growth in a different cellular setting related to this neoplasm, that is, primary cultures of mRCC cells isolated from subjects resistant to multikinase inhibitors.

## 2. Materials and Methods

### 2.1. Tumour Samples

Tumour samples were collected from 4 patients affected by metastatic renal carcinoma (mRCC) who have undergone surgical intervention or biopsy to remove metastasis (Table 1), in accordance with a protocol approved by the local Ethics Committee and after signing informed consent. We reasoned that it would be of particular interest to investigate also cells derived from primary tumour to define whether metastatic cells presented peculiar features; however, during all the observation time, we never had the availability of patients undergoing simultaneous resection of both primary tumour and metastases. So, we decided to focus this first study only on the characterization of metastatic cells. Surgical materials not required for histopathologic diagnosis were placed in sterile tubes containing RPMI 1640 supplemented with 10% heat-inactivated FBS, 200 U/mL penicillin, and 200 *μ*g/mL streptomycin (all from Life Technologies Inc., Paisley, UK).

### 2.2. Establishment of Primary Cell Cultures from Surgical Samples

Tumour samples were treated with 0.1% collagenase for half an hour and then processed with the gentle MACS Dissociator (Miltenyi Biotec, Germany), according to the manufacturer's instructions. Tumour cells were filtered (Miltenyi Biotec, Germany) to remove clusters and then collected by centrifugation at 1000 rpm for 10 minutes, checked for viability with trypan blue die exclusion, resuspended at a concentration of 0.5–1 × 10^6^ cells/mL of CellGro SCGM (Cell Genix, Freiburg, Germany), supplemented with 20% FBS, 2 mM L-glutamine, 100 U/mL penicillin, and 100 *μ*g/mL streptomycin (complete medium) (all from Life Technologies Inc.), and cultured in 25 cm^2^ tissue flasks (Corning, Stone Staffordshire, England) at 37°C and 5% CO_2_. Viable tumour cells attached to the flask within 12–24 hours. At the first medium change, rather than discarding medium containing unattached cells that may grow and provide a backup culture, we put these into a fresh flask. Cultures at 75% to 100% confluence were selected for subculture by trypsinization with 0.25% trypsin and 0.02% EDTA (Life Technologies Inc.) in a calcium/magnesium-free balanced solution. The culture medium was changed twice a week and cellular homogeneity evaluated microscopically every 24–48 hours.

To confirm the neoplastic origin of cultured cells, at least three cytospins were performed using 10^5^ cultured cells/cytospin obtained after 3–5 passages, for morphological and immunocytochemical analysis. Cells were fixed in alcohol 95°; one slide was stained with haematoxylin-eosin to identify malignant cells on the basis of cytomorphology. To distinguish tumour from hyperplastic mesothelial cells, the other slides were tested with monoclonal antibodies against cytokeratin CAM 5.2 (Dako, Glostruo, Denmark) and calretinin (Invitrogen), using indirect immunoenzymatic staining according to the manufacturers' instructions. Tumour cells were assessed by a semiquantative method as recently illustrated in [28]. In addition, since the great majority of metatastatic clear cell RCC (by far the commonest hystotype of kidney cancer) express CD10, cultured cells were also evaluated by cytofluorimetric analysis for the expression of this antigen. CD10 is usually not present on normal cells [29]. Evaluation of CD10 expression was performed by direct immunofluorescence, using phycoerythrin (PE)-anti CD10 specific monoclonal antibody (BD Pharmingen, Bioscience, Mountain View, CA), according to previously reported methods [30]. For functional experiments, tumour cells were thawed and plated at the concentration of 10–20 × 10^5^/mL and evaluated after 3-4 days when they reached the optimal confluency. In the proliferation assays, results are expressed as average number of cells (±SE) under each condition; the cells were obtained from all four patients. Differences were assessed by Student's *t*-test for unpaired values. All statistical tests were carried out with GraphPad Prism 4.

### 2.3. Isolation and Culturing of Endothelial Progenitor Cells

Since a number of drugs and agonists failed to affect intracellular Ca^2+^ dynamics in mRCC cells, we sought to verify their efficacy in another cellular setting, such as circulating EPCs isolated from healthy donors. We have recently shown that Ca^2+^ signaling is a key to EPC activation [9–11, 31, 32], so that they are suitable to serve as positive control for many substances commonly utilized to study subcellular Ca^2+^ movements [3, 25]. Blood samples (40 mL) were obtained from healthy human volunteers aged from 22 to 28 years old. The Institutional Review Board at “Istituto di Ricovero e Cura a Carattere Scientifico Policlinico San Matteo Foundation” in Pavia approved all protocols and specifically approved this study. Informed written consent was obtained according to the Declaration of Helsinki. We focused on the so-called endothelial colony forming cells (ECFCs) [33], a subgroup of EPCs which are found in the CD34^+^ CD45^−^ fraction of circulating mononuclear cells and exhibit robust proliferative potential and form capillary-like structures* in vitro* [4, 33]. To isolate ECFCs, mononuclear cells (MNCs) were separated from peripheral blood (PB) by density gradient centrifugation on lymphocyte separation medium for 30 min at 400 g and washed twice in EBM-2 with 2% FCS. A median of 36 × 10^6^ MNCs (range 18–66) was plated on collagen-coated culture dishes (BD Biosciences) in the presence of the endothelial cell growth medium EGM-2 MV Bullet Kit (Lonza) containing endothelial basal medium (EBM-2), 5% foetal bovine serum, recombinant human (rh) EGF, rhVEGF, rhFGF-B, rhIGF-1, ascorbic acid, and heparin and maintained at 37°C in 5% CO_2_ and humidified atmosphere. Discard of nonadherent cells was performed after 2 days; thereafter, medium was changed three times a week. The outgrowth of endothelial cells from adherent MNCs was characterized by the formation of a cluster of cobblestone-appearing cells [11]. That ECFC-derived colonies belonged to endothelial lineage was confirmed as described in [10, 11].

### 2.4. Solutions

Physiological salt solution (PSS) had the following composition (in mM): 150 NaCl, 6 KCl, 1.5 CaCl_2_, 1 MgCl_2_, 10 Glucose, and 10 Hepes. In Ca^2+^-free solution (0Ca^2+^), Ca^2+^ was substituted with 2 mM NaCl, and 0.5 mM EGTA was added. Solutions were titrated to pH 7.4 with NaOH. The solution was then titrated to pH 7.4 with KOH. The osmolality of the extracellular solution was 338 mmol/kg, as measured with an osmometer (Wescor 5500, Logan, UT).

### 2.5. [Ca^2+^]_*i*_ Measurements and Statistics of Ca^2+^ Signals

mRCC cells were loaded with 4 *μ*M fura-2 acetoxymethyl ester (fura-2/AM; 1 mM stock in dimethyl sulfoxide) in PSS for 1 hour at room temperature. After washing in PSS, the coverslip was fixed to the bottom of a Petri dish and the cells were observed by an upright epifluorescence Axiolab microscope (Carl Zeiss, Oberkochen, Germany), usually equipped with a Zeiss ×40 Achroplan objective (water-immersion, 2.0 mm working distance, 0.9 numerical aperture). The cells were excited alternately at 340 and 380 nm, and the emitted light was detected at 510 nm. A first neutral density filter (1 or 0.3 optical density) reduced the overall intensity of the excitation light and a second neutral density filter (optical density = 0.3) was coupled to the 380 nm filter to approach the intensity of the 340 nm light. A round diaphragm was used to increase the contrast. The excitation filters were mounted on a filter wheel (Lambda 10, Sutter Instrument, Novato, CA, USA). Custom software, working in the LINUX environment, was used to drive the camera (Extended-ISIS Camera, Photonic Science, Millham, UK) and the filter wheel and to measure and plot on-line the fluorescence from 10 up to 100 rectangular “regions of interest” (ROI). Each ROI was identified by a number. Since cell borders were not clearly identifiable, a ROI may not include the whole cell or may include part of an adjacent cell. Adjacent ROIs never superimposed. [Ca^2+^]_*i*_ was monitored by measuring, for each ROI, the ratio of the mean fluorescence emitted at 510 nm when exciting alternatively at 340 and 380 nm (shortly termed “ratio”). An increase in [Ca^2+^]_*i*_ causes an increase in the ratio [34]. Ratio measurements were performed and plotted on-line every 3 s. The experiments were performed at room temperature (22°C). All the data have been collected from mRCC cells isolated from all four patients and from EPCs harvested from three different healthy donors. The amplitude of the peak Ca^2+^ response was measured as the difference between the ratio at the peak (either of intracellular Ca^2+^ mobilization in 0Ca^2+^ or of Ca^2+^ entry occurring upon Ca^2+^ restoration to the bath) and the mean ratio of 1 min baseline before the peak. Pooled data are given as mean ± SE and statistical significance (*P* < 0.05) was evaluated by Student's *t*-test for unpaired observations.

### 2.6. RNA Isolation and Real Time RT-PCR (qRT-PCR)

Total RNA was extracted from mRCC cells derived from all the four established cultures by using the QIAzol Lysis Reagent (QIAGEN, Italy). Single cDNA was synthesized from RNA (1 *μ*g) using random hexamers and M-MLV Reverse Transcriptase (Invitrogen S.R.L., Italy). Reverse transcription was always performed in the presence or absence (negative control) of the reverse transcriptase enzyme. qRT-PCR was performed in triplicate using 1 *μ*g cDNA and specific primers (intron-spanning primers) for Stim1-2, Orai1-3, TRPC1-7, and InsP3Rs1-3, as previously described [10, 11] (Tables 2, 3, and 4). Briefly, GoTaq qPCR Mastermix (Promega, Italy) was used according to the manufacturer instruction and qRT-PCR was performed using Rotor Gene 6000 (Corbett, Concorde, NSW, Australia). The conditions were as follows: initial denaturation at 95°C for 5 min; 40 cycles of denaturation at 95°C for 30 sec; annealing at 58°C for 30 sec, and elongation at 72°C for 40 sec. The qRT-PCR reactions were normalized using *β*-actin as housekeeping gene. Melting curves were generated to detect the melting temperatures of specific products immediately after the PCR run. The triplicate threshold cycles (Ct) values for each sample were averaged resulting in mean Ct values for both the gene of interest and the housekeeping gene *β*-actin. The gene Ct values were then normalized to the housekeeping gene by taking the difference: ΔCt = Ct[gene] − Ct[*β*-actin], with high ΔCt values reflecting low mRNA expression levels. The sequences of the bands were checked by using the Big dye terminator cycle sequencing kit (Applied Biosystem, PE, USA). PCR products were also separated with agarose gel electrophoresis, stained with ethidium bromide, and acquired with the Image Master VDS (Amersham Biosciences Europe, Italy). The molecular weight of the PCR products was compared to the DNA molecular weight marker VIII (Roche Molecular Biochemicals, Italy).

### 2.7. Sample Preparation and Immunoblotting

mRCC were homogenized by using a Dounce homogenizer in a solution containing: 250 mM sucrose, 1 mM EDTA, 10 mM Tris-HCl, pH 7.6, 0.1 mg/mL PMSF, 100 mM *β*-mercaptoethanol, and Protease Inhibitor Cocktail (P8340, Sigma, USA). The homogenates were solubilized in Laemmli buffer [32] and 30 *μ*g proteins were separated on 10% SDS-polyacrylamide gel electrophoresis and transferred to the Hybond-P PVDF Membrane (GE Healthcare, Italy) by electroelution. After 1 h blocking with Tris buffered saline (TBS) containing 3% BSA and 0.1% Tween (blocking solution), the membranes were incubated for 3 h at room temperature with the following affinity purified antibodies diluted 1 : 200 in the TBS and 0.1% Tween: anti-Stim1 (sc-166840), anti-Orai1 (sc-68895), anti-TRPC1 (sc-133076), anti-TRPC3/6/7 (sc-15056), and anti-IP3R-I/II/III (sc-377518) from Santa Cruz Biotechnology, anti-Orai3 (HPA015022) and anti-Stim2 (PRS4123) from Sigma-Aldrich (Italy), and anti *β*-actin rabbit antibody as control (Rockland Immunochemicals for Research, USA; code, 600-401-886). The membranes were washed and incubated for 1 h with peroxidase-conjugated mouse, rabbit, or goat IgG (1 : 120000 in blocking solution), from Dakocytomation (P0260), Chemicon (AP132P), and Santa Cruz (sc-2354), respectively. The bands were detected with the ECL Select western blotting detection system (GE Healthcare Europe GmbH, Italy). Prestained molecular weight markers (SDS7B2, Sigma, Italy) were used to estimate the molecular weight of the bands. Control experiments were performed by using the antibody preadsorbed with a 20-fold molar excess of the immunizing peptide or by incubating the blots with nonimmune serum.

### 2.8. Protein Content

Protein contents of all the samples were determined by the Bradford's method using bovine serum albumin (BSA) as standard [35].

### 2.9. Chemicals

EBM and EGM-2 were purchased from Clonetics (Cell System, St. Katharinen, Germany). Fura-2/AM was obtained from Molecular Probes (Molecular Probes Europe BV, Leiden, the Netherlands). N-(4-[3,5-bis(trifluoromethyl)-1H-pyrazo  l-1-yl]phenyl)-4-methyl-1,2,3-thiadiazole-5-carboxamide(BTP-2) was purchased from Calbiochem (La Jolla, CA, USA). CAI was a gift from the Drug Synthesis and Chemistry Branch, National Cancer Institute (Bethesda, MD). Pyr6 has been synthesized as described in [36]. All other chemicals were obtained from Sigma Chemical Co. (St. Louis, MO, USA).

## 3. Results

### 3.1. Store-Operated Ca^2+^ Entry Is Functionally Expressed in mRCC Cells

**Figure 1:**
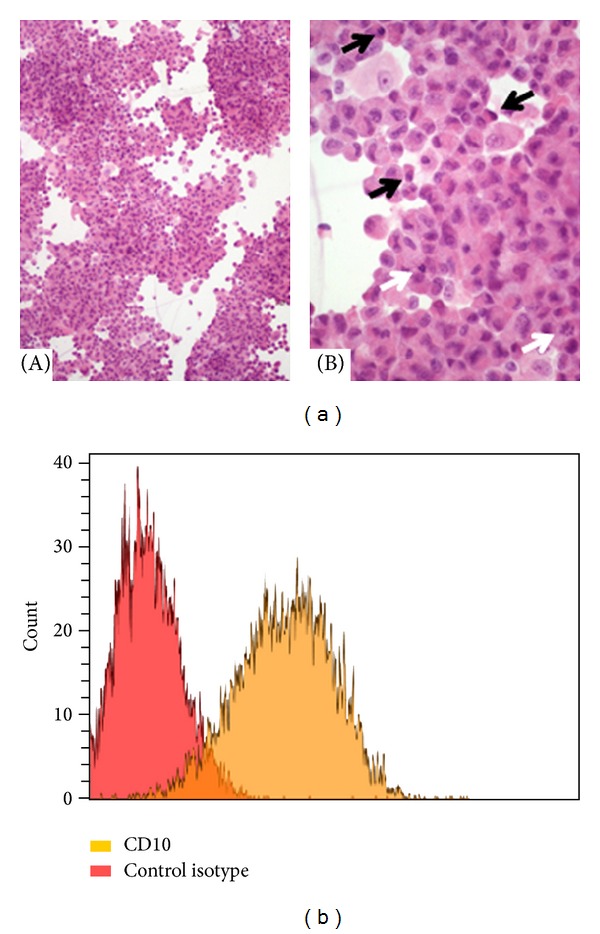
Phenotypic characterization of primary cultures of mRCC cells. (a) Morphology of mRCC cells (hematoxylin and eosin). (A) The cultured proliferation is composed of loose sheets of neoplastic cells on a scant background composed of histiocytic elements (HE, 10x); (B) at higher magnification (HE, 40x), prominent cytologic atypia is evident, consistent with pleomorphism, irregular nuclei with altered nuclear-to-cytoplasm ratio, nuclear pycnosis (black arrows), and mitoses (white arrows). (b) Expression of anti-CD10 mAb on the surface of mRCC cells as compared to the control isotype.

**Figure 2:**
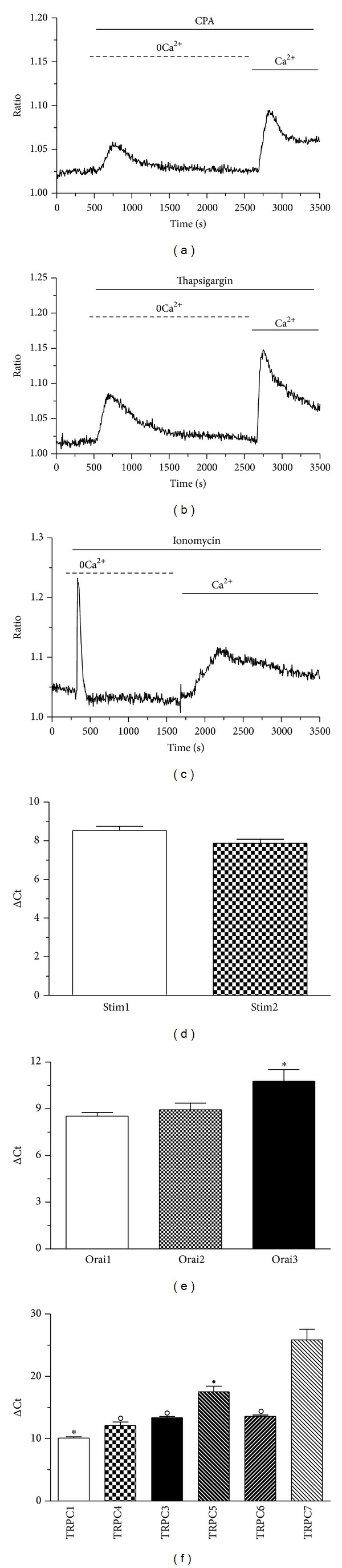
Store-operated Ca^2+^ entry is present in primary cultures of mRCC cells. The “Ca^2+^ add-back” protocol revealed that emptying the intracellular Ca^2+^ pool with CPA (10 *μ*M; (a)), thapsigargin (2 *μ*M; (b)), or ionomycin (5 *μ*M; (c)) in the absence of extracellular Ca^2+^ (0Ca^2+^) led to a robust increase in [Ca^2+^]_*i*_ upon Ca^2+^ restoration to the bath, which is the hallmark of SOCE. Quantitative real-time reverse transcription polymerase chain reaction of total RNA performed by using specific primers revealed that transcripts encoding for Stim1-2 (d), Orai1–3 (e), and TRPC1–7 (f) are expressed in mRCC cells. Bars represent the mean ± SEM of at least 4 different experiments each from different RNA extracts (see Section 2 for details). * and °*P* < 0.05 (one-way ANOVA followed by Newman-Keuls' *Q* Test).

Primary cell cultures of mRCC were performed as described in Materials and Methods section and their morphology after staining with hematoxylin and eosin is illustrated in Figure 1(a). Immunofluorescence revealed that >95% of cells were positive for CD10 staining, thereby confirming that they belonged to the neoplastic phenotype (Figure 1(b)). Store-operated Ca^2+^ entry in primary cultures of mRCC cells was, thus, evaluated by exploiting the “Ca^2+^ add-back” protocol [10]. Fura-2-loaded cells were challenged with cyclopiazonic acid (CPA) and thapsigargin, two well-known inhibitors of the Sarco-Endoplasmic Reticulum Ca^2+^ ATPase (SERCA). These drugs prevent the pump from counterbalancing the passive Ca^2+^ leak from the stores to the cytosol, thereby leading to a massive drop in the ER Ca^2+^ content which signals the gating of store-operated Ca^2+^ channels on the plasma membrane [10]. As depicted in Figures 2(a) and 2(b), both CPA (10 *μ*M) and thapsigargin (2 *μ*M) elicited a robust SOCE in mRCC cells. Similarly, SOCE was activated by ionomycin (5 *μ*M) (Figure 2(c)), a Ca^2+^-ionophore that mobilizes the entire amount of luminally stored Ca^2+^ by forming Ca^2+^-conducting pores on ER membrane. Hence, a functional SOCE is present in primary cultures of mRCC cells.

### 3.2. The Molecular Candidates to Mediate SOCE Are Expressed in mRCC Cells

**Figure 3:**
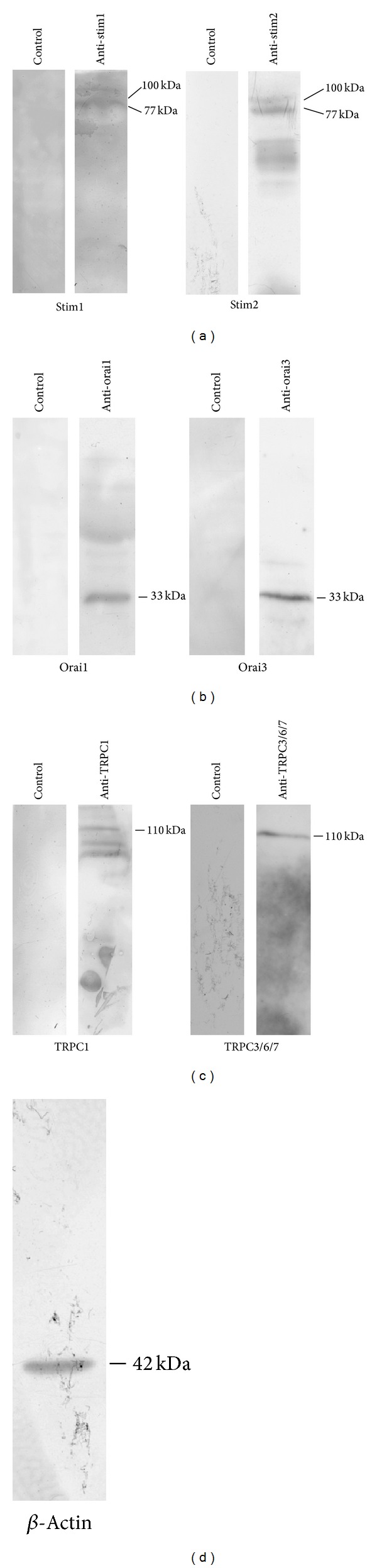
Expression of Stim1-2, Orai1, Orai3, TRPC1, and TRPC3/6/7 proteins in mRCC cells. Expression of Orai1, 3, Stim1, 2, TRPC1, and TRPC3/6/7 proteins in mRCC. Blots representative of two were shown. Lanes were loaded with 30 *μ*g of proteins, probed with affinity purified antibodies and processed as described in Section 2. Major bands of the expected molecular weights were observed. Bands were acquired with the Image Master VDS (Amersham Biosciences Europe, Italy). *β*-actin (d) has been exploited as housekeeping protein.

The putative molecular underpinnings of SOCE in mRCC cells were scrutinized by performing a qRT-PCR analysis of their total mRNA content. We focused on Stim1-2, Orai1–3, TRPC1, and TRPC3–7. TRPC2 is a pseudogene in humans and has not been investigated. All the transcripts investigated were readily detectable (Figures 2(d)–2(f)). Single bands of the expected size of cDNA fragments were amplified, as previously shown in [11]. Negative controls were performed by omitting the reverse transcriptase (not shown). The comparison of ΔCt values of the mRNAs obtained by qRT-PCR showed that Stim1 and Stim2 were equally expressed (Figure 2(d)); conversely, Orai3 expression is about fourfold less than Orai1 and Orai2 isoforms (Figure 2(e)), while TRPC3, 4, 6 levels are about threefold less than TRPC1 (Figure 2(f)). Similarly, TRPC5 is about 300 fold less expressed than TRPC1, while TRPC7 is nearly absent (Figure 2(f)). In order to confirm transcript expression at protein level, we carried out a number of western blot experiments by utilizing affinity-purified antibodies selectively targeting Stim1, Stim2, Orai1, Orai3, TRPC1, and TRPC3/6/7 [10, 31, 32]. Since TRPC7 transcript is barely expressed, the latter signal is likely to be generated by TRPC3 and TRPC6. As depicted in Figure 3, immunoblots showed a major band of about 33 kDa for Orai1 and Orai3 (Figure 3(b)), whereas Stim1 and Stim2 displayed a doublet of about 77 and 100 kDa (Figure 3(a)), and TRPC1 and TRPC3/6/7 exhibited major bands of about 110 kDa (Figure 3(c)). The band sizes were in agreement with those previously observed by using the same antibodies in circulating EPCs [10, 31, 32]. Therefore, mRCC cells are endowed with all the molecular candidates to mediate the store-dependent Ca^2+^ influx induced by CPA, thapsigargin, and ionomycin.

### 3.3. Pharmacological Profile of SOCE in mRCCs

**Figure 4:**
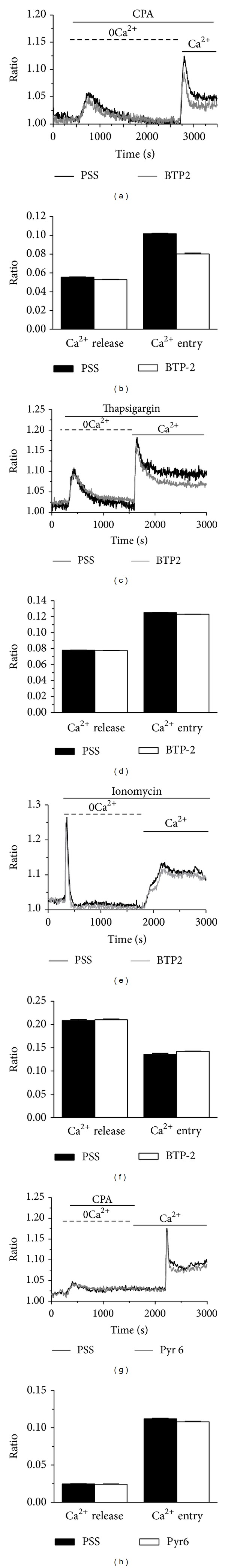
Store-operated Ca^2+^ entry is not sensitive to BTP-2 and Pyr 6 in mRCC cells. Biphasic Ca^2+^ response evoked by CPA ((a); 10 *μ*M), thapsigargin (2 *μ*M; (c)), or ionomycin (5 *μ*M; (e)) in mRCC cells pretreated with BTP-2 (20 *μ*M) for 20 min. Statistical analysis conducted on 80–90 cells per conditions revealed that BTP-2 did not affect the peak amplitudes of both Ca^2+^ release and Ca^2+^ entry stimulated by CPA (b), thapsigargin (d), or ionomycin (f). Similarly, 5 min preincubation with Pyr6 (10 *μ*M) did not affect CPA-induced intracellular Ca^2+^ discharge and SOCE in mRCC cells (g). (h) Statistical evaluation of the effect exerted by Pyr6 on the peak amplitudes of both Ca^2+^ release and Ca^2+^ entry stimulated by CPA.

**Figure 5:**
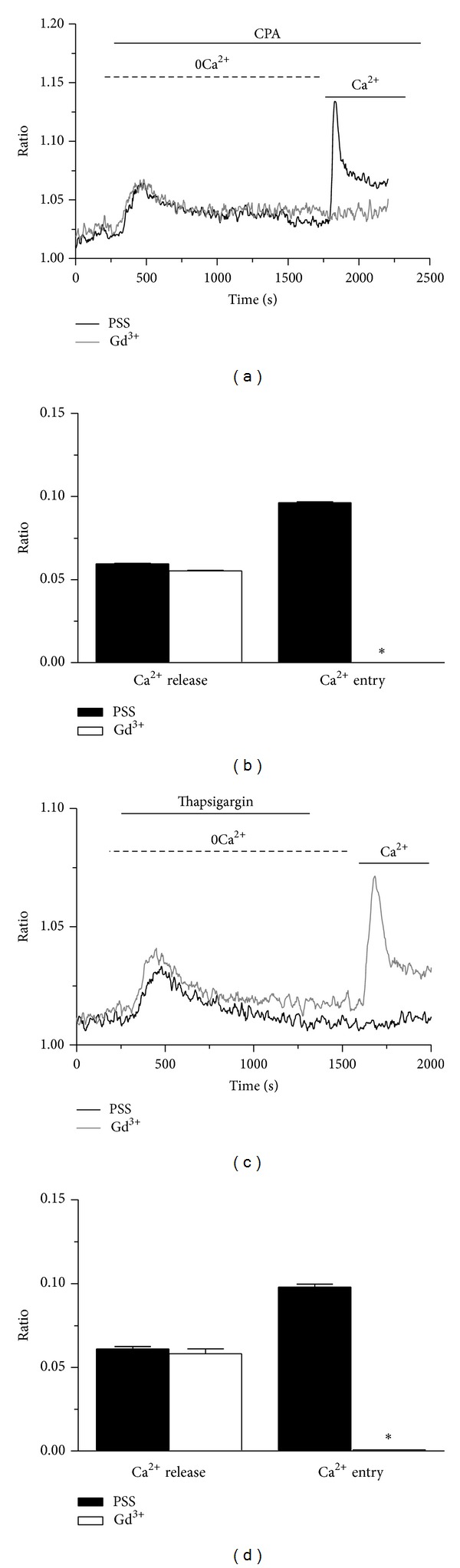
Lanthanides inhibit store-operated Ca^2+^ entry in mRCC cells. Forty minutes pretreatment with Gd^3+^ (10 *μ*M) selectively inhibited SOCE elicited by either CPA (10 *μ*M; (a)) or thapsigargin (2 *μ*M; (c)). Statistical evaluation conducted on more than 100 cells per condition confirmed that Gd^3+^ reduced SOCE, but not intracellular Ca^2+^ release, activated by CPA (b) or thapsigargin (d). *P < 0.05 (Student's *t* test).

**Figure 6:**
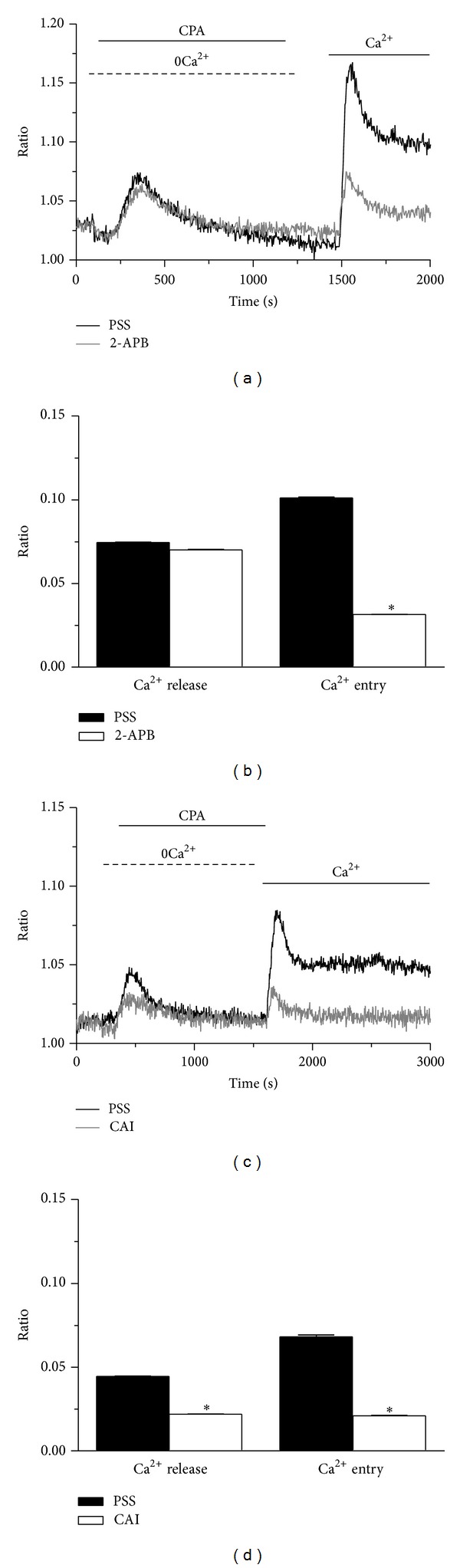
2-Aminoethoxydiphenyl borate and carboxyamidotriazole block store-operated Ca^2+^ entry in mRCC cells. (a) 2-APB (50 *μ*M, 20 min of pre-incubation) selectively inhibited CPA (10 *μ*M)-induced SOCE, but not intracellular Ca^2+^ mobilization. (b) Statistical analysis of the effect exerted by 2-APB on the peak amplitudes of CPA-induced Ca^2+^ release and Ca^2+^ entry. (c) CAI (10 *μ*M, 20 min of preincubation) reduced both phases of the Ca^2+^ response to CPA (10 *μ*M). (d) Statistical analysis of CAI-dependent inhibition of CPA-elicited Ca^2+^ release and CPA-elicited Ca^2+^ entry. *P < 0.05 (Student's *t* test).

SOCE in naïve cells may be inhibited by the pyrazole derivative BTP-2 (20 *μ*M), low concentrations of lanthanides (e.g., 1–10 *μ*M Gd^3+^), and the membrane permeable synthetic drug 2-aminoethyldiphenyl borate (2-APB) [4, 25, 31, 34]. Unexpectedly, BTP-2 (20 *μ*M; 20 min) did not affect either Ca^2+^ release or SOCE occurring in response to CPA (10 *μ*M) in mRCC cells (Figures 4(a) and 4(b)). Similarly, BTP-2 (20 *μ*M; 20 min) did not reduce the amplitude of intracellular Ca^2+^ release and extracellular Ca^2+^ inflow induced by either thapsigargin (2 *μ*M; Figures 4(c) and 4(d)) or ionomycin (5 *μ*M; Figures 4(e) and 4(f)). These results strongly suggest that neither Orai1 nor TRPC1, which are dramatically sensitive to this drug, contribute subunits to the conducting pore of store-dependent channels in mRCC cells [3, 4]. Then, we probed the effects of 10 *μ*M Gd^3+^, which selectively abrogates Ca^2+^ entry through Orai1 and TRPC1 [3]. Our preliminary experiments demonstrated that preincubating the cells for 30 min with 10 *μ*M Gd^3+^ did not produce any significant reduction in both CPA- and thapsigargin-induced SOCE (*n* = 84 and *n* = 96, resp.; data not shown). Likewise, Pyr 6 (10 *μ*M, 5 min), a recently synthesized specific Orai1 blocker did not affect the biphasic Ca^2+^ response to CPA (*n* = 82; Figures 4(g)-4(h)). Therefore, we turn on to 100 *μ*M Gd^3+^, which exerts an unspecific inhibition on TRPC-gated Ca^2+^ inflow. At this dose, Gd^3+^ suppressed SOCE in response to either CPA (Figures 5(a) and 5(b)) or thapsigargin (Figures 5(c) and 5(d)), without affecting intracellular Ca^2+^ release (Figures 4(a)–4(d)). Then, we ascertained the inhibitory action of 2-APB, a largely utilized inhibitor of both Orai- and TRPC-gated Ca^2+^ entry [3]. We found that 2-APB (50 *μ*M) selectively reduced CPA-induced SOCE (Figures 6(a) and 6(b)). Finally, we focused on CAI, a synthetic small molecule inhibitor of non-voltage-gated Ca^2+^ channels which was already tested on mRCC patients within a phase II clinical trial, but eventually failed [3]. Twenty min pretreatment with 20 *μ*M significantly reduced CPA-evoked intracellular Ca^2+^ release and SOCE (Figures 6(c) and 6(d)). Overall, these data show that the pharmacology of SOCE in mRCC cells is rather unusual, such pathway being insensitive to BTP-2, low concentrations of lanthanides, and Pyr6, while it was blocked by 100 *μ*M Gd^3+^, 2-APB, and CAI. As discussed in more detail below, this profile is consistent with a Ca^2+^-conducting pore constituted by the heteromeric interaction of multiple TRPC and, perhaps, Orai subunits.

### 3.4. Weak InsP_3_-Dependent Ca^2+^ Signalling in mRCCs

**Figure 7:**
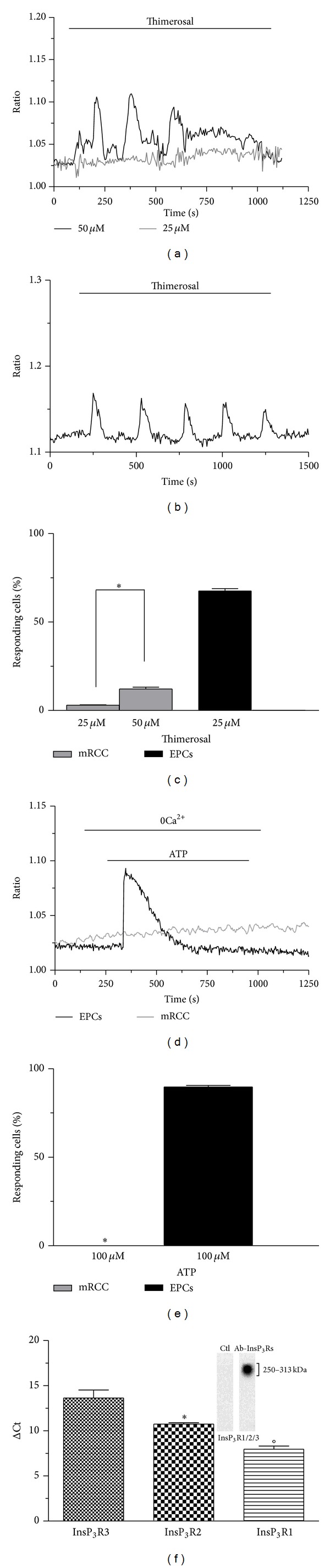
InsP_3_-dependent signalling in mRCC cells and endothelial progenitor cells. (a) Thimerosal triggered a brief train of intracellular Ca^2+^ spikes in mRCC cells when applied at 50 *μ*M (black tracing), while it was almost ineffective at 25 *μ*M (grey tracing). (b) Prolonged oscillations in intracellular Ca^2+^ levels stimulated by thimerosal (same solution as that used in (a), recording performed on the same day) in EPCs isolated from mRCC patients. (c) Percentage of mRCC cells and EPCs responding to thimerosal. *P < 0.05 (Student's *t*-test). (d) 100 *μ*M ATP stimulated InsP_3_-dependent Ca^2+^ mobilization from EPCs (black tracing), but not from mRCC cells (grey tracing). The same solution was used in both experiments and the recordings were conducted on the same day. (e) Fraction of mRCC cells and EPCs responding to ATP. *P < 0.05 (Student's *t*-test). (f) InsP_3_R transcripts in primary mRCC cells. *P < 0.05 versus InsP_3_R1, °*P* < 0.05 versus InsP_3_R2. The inset illustrates InsP_3_R expression detected by an InsP_3_R-I/II/III antibody. A major band that corresponds to the sum of the three single 313/260/250 kDa bands for InsP_3_R1/2/3 was found.

**Figure 8:**
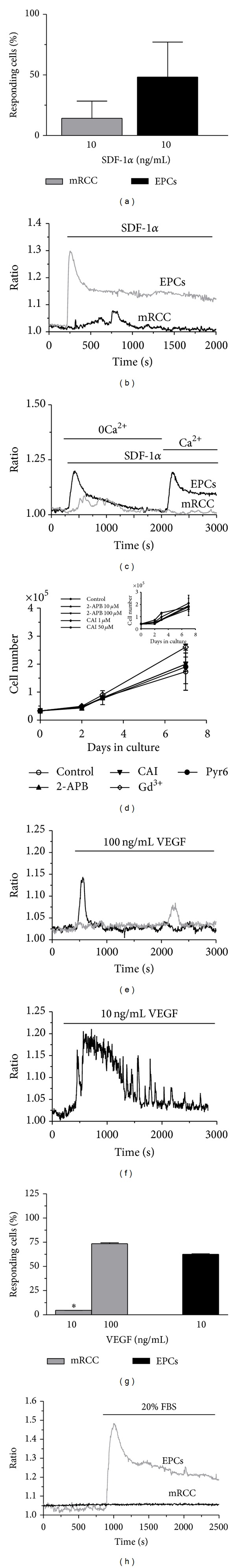
Stromal derived factor 1-*α* (SDF-1*α*), vascular endothelial growth factor (VEGF), and foetal bovine serum (FBS) do not trigger store-operated Ca^2+^ entry in mRCC cells. (a) fraction of cells responding to SDF-1*α* in mRCC cells and EPCs. (b) SDF-1*α* (10 ng/mL), produced a Ca^2+^ transient (black tracing) in mRCC cells while it elicited a biphasic increase in [Ca^2+^]_*i*_, which is the hallmark of SOCE activation, in EPCs. (c) SOCE was triggered by SDF-1*α* (10 ng/mL) in EPCs, but not in mRCC cells. (d) 2-APB (50 *μ*M), CAI (10 *μ*M), Gd^3+^ (100 *μ*M) did not inhibit mRCC cell proliferation. Inset, lower, and higher doses of 2-APB and CAI did not impair mRCC cell proliferation. (e) Brief elevation in [Ca^2+^]_*i*_ induced by VEGF (100 ng/mL) in mRCC cells. (f) VEGF (10 ng/mL) ignites repetitive Ca^2+^ oscillations in EPCs. (g) Fraction of cells responding to VEGF in mRCC cells and EPCs. (h) 20% foetal bovine serum (FBS) elicits a biphasic elevation in [Ca^2+^]_*i*_ in EPCs, but not mRCC cells.

Then, we sought to assess whether InsP_3_ signalling is coupled to SOCE activation in mRCC cells. The thiol-reactive agent thimerosal, which serves as InsP3R agonist in a variety of cell types, has widely been employed to stimulate InsP3-dependent Ca2+ release [37]. However, 25 *μ*M thimerosal was ineffective in the large majority of cells tested (Figures 7(a) and 7(c)), while it induced a brief burst of Ca^2+^ spikes only in a modest fraction of cells when its concentration was raised up to 50 *μ*M (Figures 7(b) and 7(c)). In order to assess whether the drug was indeed capable of stimulating InsP_3_-dependent Ca^2+^ release, we probed its effects on EPCs isolated from healthy donors, which served as positive control. Twenty-five *μ*M thimerosal evoked repetitive oscillations in intracellular Ca^2+^ concentration ([Ca^2+^]_*i*_) in ~70% of cells (Figures 7(b) and 7(c)), which testify for the efficacy of the drug batch we employed. This issue was further investigated by challenging mRCC cells with the InsP_3_-synthesizing transmitter, ATP, which binds to purinergic P_*2Y*_ receptors to engage PLC-*β* and activate SOCE [10, 11]. As shown in Figure 7(d) (grey tracing) and Figure 7(e), 100 *μ*M ATP was never able to induce Ca^2+^ mobilization from ER in mRCC cells, albeit in parallel experiments it induced InsP_3_-dependent Ca^2+^ discharge and SOCE in most EPCs (black tracing in Figures 7(d) and 7(e)). These results could be explained by the lack of InsP_3_Rs in mRCC cells. However, qRT-PCR analysis unveiled that these cells are present with the transcripts encoding for all the three known isoforms of InsP_3_Rs (i.e., InsP_3_R1–3; Figure 7(f)) [37], the relative pattern of expression being InsP_3_R3 > InsP3R2 > InsP3R1. InsP_3_R expression was further probed by immunoblotting, which revealed a large band, deriving from the sum of 313/260/250 kDa bands, for InsP_3_R1/2/3. These data were confirmed by administrating SDF-1*α* (10 ng/mL), the most important chemoattractant cytokine involved in cancer dissemination, which induces SOCE upon InsP_3_-dependent mobilization of intraluminally stored Ca^2+^ [38]. SDF-1*α* (10 ng/mL) elicited a transient elevation in [Ca^2+^]_*i*_ in a minor proportion of mRCC cells (Figures 8(a) and 8(c)). This pattern of Ca^2+^ signalling is consistent with a modest intracellular Ca^2+^ release, but not with a sizeable Ca^2+^ influx [16]. Conversely, SDF-1*α* (10 ng/mL) elicited a rapid Ca^2+^ peak which rapidly decayed to a plateau phase of intermediate amplitude, which is the typical hallmark of SOCE [16], in EPCs (Figures 8(a) and 8(b)). Accordingly, the “Ca^2+^ add-back” protocol revealed that SDF-1*α*-induced SOCE was present in these cells, but not in mRCC cells (Figure 8(c)). Taken together, these data demonstrate that InsP_3_ signalling is rather weak and might be not tightly coupled to SOCE in mRCC cells.

### 3.5. Store-Operated Ca^2+^ Entry Does Not Control Proliferation in mRCC Cells

**Figure 9:**
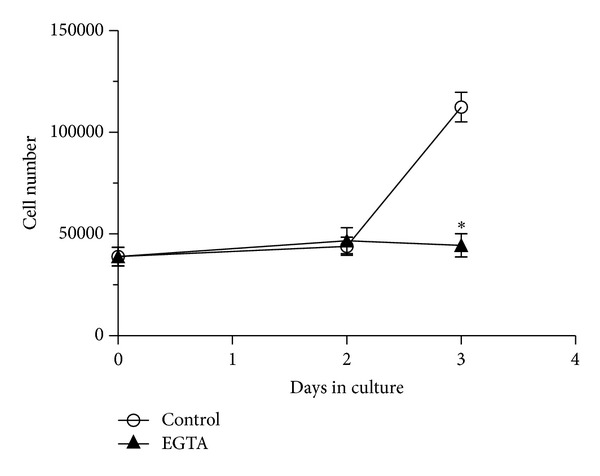
Chelation of extracellular Ca^2+^ blocks proliferation in mRCC cells. Removal of extracellular Ca^2+^ by supplementing the culture medium with 5 mM EGTA prevents mRCC cells from proliferating. *P < 0.05 (Student's *t*-test).

The metastatic RCC cells utilized in the present study have been isolated from malignant lesions and have, thus, already been exposed to the chemotactic clues that drive them to the target organ from the primary tumour. In the search for an alternative target to eradicate disseminated metastases from the patients, we reasoned it was more appropriate to focus on SOCE involvement in mRCC proliferation. Consequently, we probed the effect of 100 *μ*M Gd^3+^, 50 *μ*M 2-APB, 20 *μ*M CAI, and 10 *μ*M, as negative control, on mRCC proliferation.  Figure 8(d) clearly shows that none of these compounds inhibited cell growth after 7 days in culture; therefore, SOCE is not required in the replication process in these cells. Among the signalling pathways utilized by growth factors to promote cell cycle progression, store-dependent Ca^2+^ inflow stands out as a prominent mechanism [4, 13, 14, 25, 39]. In agreement with the lack effect of SOCE inhibition on mRCC cell proliferation, 20% FBS did not generate any detectable increase in [Ca^2+^]_*i*_ (*n* = 65, data not shown). Moreover, 10 ng/mL VEGF elicited a modest Ca^2+^ transient in a small fraction of cells (Figures 8(e) and 8(g)), while it induced robust intracellular Ca^2+^ oscillations in EPCs (Figures 8(f) and 8(g)) due to the concerted interaction of InsP_3_-dependent Ca^2+^ release and SOCE [9]. The fraction of responding mRCC cells augmented when VEGF concentration was raised up to 100 ng/mL (Figure 8(g)), but the Ca^2+^ signal still lacked a plateau phase (not shown). Similarly, 20% FBS activated a biphasic Ca^2+^ signal in EPCs (grey tracing in Figure 8(h); *n* = 55), but not in mRCC cells (black tracing in Figure 8(h); *n* = 62). Finally, EGF did not ignite any detectable Ca^2+^ activity when administrated at 10 ng/mL (*n* = 74, respectively; data not shown), which has been shown to trigger a sustained Ca^2+^ inflow in other cell types [40]. Overall, these data argue against the requirement for SOCE to drive proliferation in mRCC cells. We finally sought to ascertain whether extracellular Ca^2+^ inflow is involved in mRCC cell growth. The cells were plated in complete culture medium and, after three days, external Ca^2+^ was chelated by the addition of 5 mM EGTA. As depicted in Figure 9, this treatment dramatically prevented mRCC from replicating. Therefore, albeit SOCE does not control this process, mRCC proliferation requires extracellular Ca^2+^ entry.

## 4. Discussion

Store-operated Ca^2+^ entry is among the most widespread mechanisms of Ca^2+^ entry in cancer cells, thereby contributing to regulate a growing number of processes involved in malignant transformation, including cell proliferation, differentiation, and metastatization [4, 12–14, 25, 27, 39, 41–43]. It is, therefore, not surprising that SOCE has been put forward as a promising target to develop alternative therapies of metastatic tumours. We have recently found that the pharmacological blockage of SOCE suppresses cell proliferation in circulating EPCs isolated from naïve mRCC patients [10]. Therefore, we hypothesized that SOCE inhibition could provide an alternative strategy to promote eradicate metastases in subjects resistant to multikinase inhibitors [3]. To reinforce this concept, we undertook the present investigation to assess whether SOCE drives proliferation in primary cultures of mRCC cells isolated from these patients.

The “Ca^2+^ add-back” manoeuvre revealed that a functional SOCE is present in mRCC cells and is activated upon pharmacological emptying of ER Ca^2+^ content [10, 11]. Growing body of evidence indicates that SOCE in cancer cells is mediated by Stim1, which relays the information relative to the drop in ER Ca^2+^ levels to cell periphery, where it binds to and activates Orai1, a highly Ca^2+^-selective pore-forming unit [10, 13, 14, 17, 20, 21, 42, 44]. Alternatively, SOCE might require the involvement of a less Ca^2+^-selective membrane pathway, such as that provided by TRPC channels. In more detail, all TRPC channels may participate in SOCE either through direct binding to Stim1 (i.e., TRPC1, TRPC4, and TRPC5) or upon multimerization with Stim1-gated isoforms (i.e., TRPC3 and TRPC6). In particular, TRPC1 and TRPC6 have been involved in SOCE-mediated proliferation and cytokinesis in a number of cancer types [18, 22–24, 42, 45]. The scenario becomes even more complicated when considering that Orai3 is activated by Stim1 upon intracellular store depletion in some oestrogen receptor-positive breast cancer lines [21], and that Orai1 may assemble into a supramolecular ternary complex with Stim1 and TRPC1 [46]. In the present investigation, we utilized qRT-PCR analysis to demonstrate that mRCC cells possess all the molecular candidates to mediate SOCE, that is, Stim1-2, Orai1–3, TRPC1, TRPC4, TRPC5, and TRPC6. However, Orai3 is far less expressed than Orai1 and Orai2, whereas TRPC1 is more abundant than TRPC3, TRPC4, TRPC5, and TRPC6. Immunoblotting has confirmed that mRCC cells present with Stim1-2, Orai1, Orai3, TRPC1, and TRPC6 proteins. It is worth noting that mRCC cells present with TRPC3 and TRPC5, which are absent in normal renal epithelium and in RCC cell lines [47]. This feature supports the notion that established long term cell cultures may not truly represent the biological complexity of a real metastatic phenotype. These expression data hint at the participation of Stim1-2, Orai1-2, and TRPC1 to SOCE activation in mRCC cells. However, the pharmacological profile displayed by SOCs in these cells is not entirely consistent with this hypothesis. First, BTP-2 does not affect the magnitude of store-dependent Ca^2+^ influx induced by pharmacological depletion of ER in mRCC cells. This result was somehow surprising: we had previously shown that 20 *μ*M BTP-2 fully blocks CPA- and ATP-induced SOCE in different types of EPCs [10], whereas 10–30 *μ*M BTP-2 inhibits store-dependent Ca^2+^ inflow in several other cell types [3]. As discussed elsewhere [3], BTP-2 may interfere with SOCs containing either Orai1 or TRPC1 alone or is formed by Orai1 in conjunction with TRPC1. As a consequence, these mechanisms are unlikely to be the main actors of SOCE in mRCC cells. This hypothesis is further supported by the lack of effect of 10 *μ*M Gd^3+^: at this concentration, lanthanides fully block SOCE either when it is driven by an Orai1-lined channel pore [3] or when both Orai1 and TRPC1 are part of the Ca^2+^-conducting pathway [10, 11]. Therefore, we do believe that neither Orai1 nor an Orai1-TRPC1 complex is involved in SOCE in mRCC cells. Consistently, Pyr6, a specific Orai1 inhibitor, failed to inhibit CPA-induced Ca^2+^ inflow. Conversely, SOCE is suppressed by high micromolar concentrations of Gd^3+^ and/or to 2-APB, which affect other members of the TRPC subfamily, such as TRPC3–6 [3, 4]. The wealth of information on the pharmacology of naive SOCs contributed by naïve TRPC channels is rather scarce. Both TRPC3 and TRPC6 are blocked by 2-APB [3, 48]. Nevertheless, Ca^2+^ entry through ectopically expressed TRPC3 is abrogated by lanthanides already at 10 *μ*M, while TRPC6 inhibition requires doses higher than 50 *μ*M [48]. This feature rules out TRPC3 from the candidates to mediate SOCE in mRCC cells. TRPC4 and TRPC5 are potentiated, rather than impaired, by 100 *μ*M La^3+^ and Gd^3+^ and are unaffected by 2-APB [49]. Overall, these observations are consistent with a role of TRPC6 in SOCE in mRCC cells. This hypothesis is corroborated by two pieces of evidence. First, endogenous TRPC6 mediates SOCE and sustains proliferation in Hep G2 and Huh-7 human hepatoma cells [18, 19], in human and rat glioma cell lines [50] and in human gastric cancer cells [51]. Second, TRPC6 expression is enhanced in a variety of primary neoplasms as compared to normal paratumour tissues [50–52], including RCC [53]. Third, TRPC6 is sensitive to CAI in Hep G2 and Huh-7 human hepatoma cells [19], albeit this drug may also prevent intracellular Ca^2+^ mobilization in mRCC cells. The effect exerted by CAI both on Ca^2+^ release and on store-dependent Ca^2+^ influx is a consequent to the inhibition of mitochondrial Ca^2+^ uptake, as shown in HEK293 cells [54]. Despite the pharmacological and molecular evidence of TRPC6 involvement in SOCE, we cannot rule out the contribution of Orai proteins or other TRPC channels, as part of TRPC6-containing complexes [15, 46]. For instance, TRPC4 would be indispensable to confer store sensitivity to TRPC6 [15], which is otherwise gated by diacylglycerol [48]. Moreover, over-expressed Orai1 proteins may assemble with endogenous TRPC channels in a Stim1-dependent manner to form a SOCE pathway which is insensitive to low micromolar Gd^3+^ [55]. Therefore, further studies are required to untangle the molecular architecture of SOCE in mRCC cells.

Store-operated Ca^2+^ entry is normally activated upon InsP_3_-dependent depletion of the intracellular Ca^2+^ stores [4]. A peculiar feature of SOCE in mRCC cells was its weak coupling to InsP_3_ signalling. These cells are endowed with all the three known InsP_3_R isoforms, their pattern of mRNA expression being InsP_3_R3 > InsP_3_R2 > InsP_3_R1, which is similar to that found in GMB [56] and breast carcinoma [57]. Our results revealed that InsP_3_-dependent Ca^2+^ release is loosely coupled to SOCE. Thimerosal oxidizes thiols to form a thiomercurylethyl complex, thereby sensitizing InsP_3_ to basal InsP_3_ levels [37]. In our hands, up to 50 *μ*M thimerosal was almost ineffective at mobilizing intracellular Ca^2+^ in mRCC cells, while it induced repetitive Ca^2+^ oscillations in EPCs. These observations suggest that the resting concentration of InsP_3_, which is attributable to constitutive PLC activity, is low. When we sought to physiologically increase intracellular InsP_3_ levels, we found that both ATP and SDF-1*α* fail to reproducibly mobilize intracellularly stored Ca^2+^ in mRCC cells; the same results were obtained when PLC*γ* was recruited to the plasma membrane by stimulating TKRs with either VEGF, EGF, or FBS (as discussed below). ATP, FBS, and EGF were actually unable to induce Ca^2+^ release from ER in the totality of the cells probed. It appears that mRCC cells are reluctant to generate InsP_3_-dependent Ca^2+^ signals and to activate SOCE. Indeed, both VEGF and SDF-1*α* trigger transient elevations in [Ca^2+^]_*i*_ which lack the plateau phase caused by persistent SOCE activation. InsP_3_ metabolism may be dramatically deranged in cancer cells. It has recently been demonstrated that the activity of the InsP_3_ producing enzymes, phosphatidylinositol (PI) 4-kinase, PI 4-phosphate-5-kinase, and PLC, is enhanced in neoplastic tissues, while the catabolic enzymes, PIP_2_ 5-phosphatase and PIP 4-phosphatase, are downregulated; this is likely to lead to an amplification of InsP_3_-related pathways [58]. It could be hypothesized that the opposite occurs in mRCC cells and is responsible for the weak InsP_3_ signalling we observed. An alternative, but not mutually exclusive, explanation includes the downregulation of the membrane receptors we have tried to exploit or their lower sensitivity to ligand binding in these cells (see below).

The present study aimed to assess whether the pharmacological blockade of SOCE leads to the inhibition of primary cultures established from metastatic lesions of patients resistant to the most recent anti-angiogenic therapies. Nevertheless, the inhibition of SOCE with Gd^3+^, 2-APB, and CAI did not impair mRCC cell proliferation. The lack of effect of CAI deserves a careful evaluation. This is a synthetic small molecule compound that inhibits a Ca^2+^-mediated cellular responses by exerting a nonspecific block on Ca^2+^ entry and release pathways in both malignant cells and tumour vessels [4, 25]. CAI is currently under investigation as an orally administered tumouristatic and antiangiogenic agent in clinical phase I–III trials of several solid cancers [25], including RCC [4, 25]. As reviewed in [25], this agent was supposed to possess antitumour activity against mRCC based on the observation, coming from phase I trials, of cases of disease stabilization and minor responses. Unfortunately, a randomized discontinuation trial of CAI in mRCC patients was not successful, the compound being inactive and not well tolerated. The present investigation permits understanding the failure of CAI administration at cellular level as this drug did not affect mRCC cell proliferation in our hands. To the best of our knowledge, this is the first report that CAI does not hamper cell cycle progression in tumour cells [41]. SOCE is the ubiquitous mechanism whereby Ca^2+^ inflow drives cell cycle progression and DNA synthesis in both normal and cancer cells. The “habituation” of mRCC cells to reduced SOCE is, therefore, the most remarkable finding of the present study. This feature renders RCC cells, which are freshly isolated from metastatic effusions and maintained in culture for less than 3-4 passages, different from the commercially available immortal tumour cell lines, which heavily rely on SOCE to proliferate [18–23, 44, 45, 50, 51]. Curiously, a recent investigation conducted on primary cultures established from GBM disclosed that Stim1/Orai1 account for ~20% of cell growth [17], thereby confirming that freshly isolated tumour cells may become independent on SOCE to proliferate. It is reasonable to speculate that the sharp fall in SOCE requirement to proliferate may be considered a novel mechanism of tumour resistance to pharmacological treatments that could be identified only in patients-derived cells. Consistent with these results, recent work has convincingly demonstrated that external Ca^2+^ entry through either store-operated or arachidonate-activated channels does not support proliferation in three different tumoural cell lines, such as HEK293, HeLa, and Huh-7 cells [59], and in EPCs harvested from patients affected by primary myelofibrosis [32]. In order to explain our result, it is mandatory to recall that mRCC cells have been cultured from metastatic lesions of patients who progressed after being exposed to at least two VEGF/VEGF(Rs) inhibitors and one mTOR inhibitor. These drugs are administrated to disrupt the vascular network which sustains the exceeding tumour growth and paves the ways for the dissemination of malignant deposits throughout the organism [3]. The physiological stimulus that activates SOCE to control cell proliferation is the InsP_3_-dependent drop in ER Ca^2+^ levels which is consequent to TKR stimulation by extracellular growth factors [9, 23, 45, 50]. The prolonged exposure to multikinase inhibitors, which target the intracellular pathways downstream VEGF, PDGF, and EGF receptors, might “silence” the ability of metastatic cells to respond to endogenous growth factors [60], albeit the treatment was designed to impair endothelial signalling. As a consequence, either the reduced sensitivity to ligand binding or the inhibition of TKR activation would fail to mobilize a sufficient amount of Ca^2+^ from the InsP_3_-sensitive store. Future work is required to assess whether store-dependent Ca^2+^ inflow governs cell proliferation in mRCC cells isolated from naïve patients, that is, who are yet to undergo any pharmacological treatment. Accordingly, EPCs isolated from these subjects cannot expand in the presence of SOCE inhibitors [10]. Two pieces of observations should, however, be borne in mind in regard to the role of Ca^2+^ signalling in mRCC cell growth. First, although the influx of Ca^2+^ is not important to drive proliferation in HEK293, HeLa, and Huh-7 cells, the same investigation unveiled that the genetic suppression of Orai1 and Orai3 impeded mitosis progression in these settings [59]. Therefore, these protein channels fulfill cellular functions other than conducting signalling ions across the plasma membrane that are relevant to cell proliferation [59, 61]. Second, whereas the pharmacological blockade of SOCE did not cause any significant decrease in mRCC cell number after 5 days of incubation, buffering external Ca^2+^ with EGTA blocked cell proliferation. As a consequence, this process is controlled by a different Ca^2+^ entry pathway(s) and these cells do not habituate to extracellular Ca^2+^ deprivation [26]. A variety of additional Ca^2+^-permeable may control cell growth in nonexcitable cells, such as receptor-operated channels (ROCs), second messengers-operated channels (SMOCs), voltage operated-channels (VOCs), and ionotropic receptors [16]. One or more of these conductances are likely to regulate mRCC cell proliferation and should be identified in future experiments.

## 5. Conclusions

This investigation demonstrated for the first time that a functional SOCE is present in primary cultures of mRCC cells isolated from patients treated with multikinase inhibitors. The pharmacological characterization and the molecular screening of Stim, Orai, and TRPC transcripts are consistent with the involvement of TRPC6. A role for Stim, Orai, and other TRPC isoforms cannot, however, be ruled out: in fact, these proteins tend to assemble in supramolecular heteromeric complexes which display different pharmacological, ion selectivity, and gating properties as those observed when ectopically expressed as single units. SOCE is loosely coupled to InsP_3_-dependent signalling and cannot be activated by ATP, SDF-1*α*, VEGF, FBS, and EGF. Consistent with this observation, the pharmacological blockade of SOCE does not affect mRCC proliferation. These results suggest that SOCs are unlikely to provide a suitable molecular target to design alternative treatments for subjects resistant to multikinase inhibitors. Caution is, therefore, warranted when SOCE inhibition is put forward as a novel option for cancer therapy exclusively on the basis of results obtained from immortal tumour cell lines.

## Figures and Tables

**Table 1:** Clinical characteristics of renal cellular carcinoma patients involved in this study.

Patient	Sex	Day of birth	Year of diagnosis	Histology	Stage^1^ at diagnosis	Surgery for localized disease	Systemic treatments received
LA	M	14/11/1944	1995	Clear cell RCC	pT2, Nx, M0	Yes, radical nephrectomy	IL-2 Sorafenib Sunitinib Everolimus Experimental vaccine

DPV	M	21/05/1953	2010	Bellini's duct RCC	pT2, N2, M1	Yes. Radical nephrectomy	Temsirolimus Sorafenib Sunitinib Cytotoxic chemotherapy Experimental vaccine

LG	F	25/12/1948	2005	Clear cell RCC	pT2, N0, M0	Yes. Partial nephrectomy	Cytotoxic chemotherapy Sunitinib Everolimus Sorafenib Interferon-*α* Experimental vaccine

GG	M	20/10/1959	2009	Papillary type I RCC	pT3b, N0, M1	Yes, radical nephrectomy	Sunitinib Sorafenib Everolimus Cytotoxic chemotherapy Experimental vaccine

^1^Stage is indicated according to the 2002 TNM staging system.

**Table 2:** Primer sequences used for real time reverse transcription/polymerase chain reaction of Orai1–3 and Stim1-2.

Gene	Primer sequences		Size (bp)	Accession number
Orai1	Forward	5′-AGTTACTCCGAGGTGATGAG-3′	257	NM_032790.3
	Reverse	5′-ATGCAGGTGCTGATCATGAG-3′		
Orai2	Forward	5′-CCATAAGGGCATGGATTACC-3′	334	NM_001126340.1 variant 1
	Reverse	5′-CAGGTTGTGGATGTTGCTCA-3′		NM_032831.2 variant 2
Orai3	Forward	5′-CCAAGCTCAAAGCTTCCAGCC-3′	159	NM_152288.2
	Reverse	5′-CAAAGAGGTGCACAGCCACCA-3′		
Stim1	Forward	5′-CCTCAGTATGAGGAGACCTT-3′	347	NM_003156.3
	Reverse	5′-TCCTGAAGGTCATGCAGACT-3′		
Stim2	Forward	5′-AAACACAGCCATCTGCACAG-3′	186	NM_020860.2
	Reverse	5′-GGGAAGTGTCGTTCCTTTGA-3′		
*β*-actin	Hs_ACTB_1_SG, QuantiTect Primer Assay QT00095431, Qiagen		146	NM_001101

**Table 3:** Primer sequences used for real time reverse transcription/polymerase chain reaction.

Gene	Primer sequences		Size (bp)	Accession number
TRPC1	Forward	5′-ATCCTACACTGGTGGCAGAA-3′	307	NM_003304.4
	Reverse	5′-AACAAAGCAAAGCAGGTGCC-3′		
TRPC3	Forward	5′-GGAGATCTGGAATCAGCAGA-3′	336	NM_001130698.1 variant 1
	Reverse	5′-AAGCAGACCCAGGAAGATGA-3′		NM_003305.2 variant 2
TRPC4	Forward	5′-ACCTGGGACCTCTGCAAATA-3′	300	NM_016179.2 variant alpha
	Reverse	5′-ACATGGTGGCACCAACAAAC-3′		NM_001135955.1 variant beta
				NM_001135956.1 variant gamma
				NM_001135957.1 variant delta
				NM_003306.1 variant epsilon
				NM_001135958.1 variant zeta
TRPC5	Forward	5′-GAGATGACCACAGTGAAGAG-3′	221	NM_012471.2
	Reverse	5′-AGACAGCATGGGAAACAGGA-3′		
TRPC6	Forward	5′-AGCTGTTCCAGGGCCATAAA-3′	341	NM_004621.5
	Reverse	5′-AAGGAGTTCATAGCGGAGAC-3′		
TRPC7	Forward	5′-CACTTGTGGAACCTGCTAGA-3′	387	NM_020389.1
	Reverse	5′-CATCCCAATCATGAAGGCCA-3′		

**Table 4:** Primer sequences used for real time reverse transcription/polymerase chain reaction of InsP_3_ receptors.

Gene	Primer sequences		Size (bp)	Accession number
InsP_3_R1	Forward	5′-TCAACAAACTGCACCACGCT-3′	180	ENSG00000150995
	Reverse	5′-CTCTCATGGCATTCTTCTCC-3′		
InsP_3_R2	Forward	5′-ACCTTGGG GTTAGTGGATGA-3′	158	ENSG00000123104
	Reverse	5′-CCTTGTTTGGCTTGCTTTGC-3′		
InsP_3_R3	Forward	5′-TGGCTTCATCAGCACTTTGG-3′	173	ENSG00000096433
	Reverse	5′-TGTCCTGCTTAGTCTGCTTG-3′		
*β*-actin	Hs_ACTB_1_SG, QuantiTect Primer Assay QT00095431, Qiagen		146	NM_001101
